# Trial history shapes the alerting-congruency interaction in selective attention

**DOI:** 10.3389/fpsyg.2026.1768354

**Published:** 2026-06-11

**Authors:** Maya J. Golden, Thomas G. Hutcheon, Carlos Gomez-Rivas, Todd A. Kahan

**Affiliations:** 1 Department of Psychology, Bates College, Lewiston, ME, United States; 2 Psychology Program, Bard College, Annandale-on-Hudson, NY, United States

**Keywords:** cognitive control, phasic alertness, selective attention, stimulus–response priming, working memory capacity

## Abstract

Cues that alert a person to the presence of an upcoming stimulus increase phasic alertness and speed responses, but these cues can come at a cost where distractors have a greater influence on performance. The interaction between alerting and distractor congruency reveals how transient increases in alertness influence performance. This study investigated whether trial history influences this interaction. We tested whether the interaction between alerting and congruency was moderated by the congruency of the preceding trial or by response repetitions. Using a large online sample (*N* = 165), participants completed an arrow-flanker task with and without alerting cues along with an Automated Operation Span Task (AOSPAN) to assess working memory capacity (WMC). Results replicated the alerting-congruency interaction, showing that alerting cues amplified distractor interference. The alerting-congruency interaction was not moderated by preceding congruency in either reaction times or accuracy. In contrast, the alerting-congruency interaction was moderated by response repetition in accuracy (but not response time). Working memory capacity was not associated with the alerting-congruency interaction. Together, this pattern is most consistent with the claim that alerting cues boost phasic arousal which amplifies prepotent stimulus–response associations.

## Introduction

Researchers have identified three attentional networks (alerting, cognitive control, and orienting) that help us prioritize important stimuli and ignore irrelevant information (Fan et al., 2002; Posner, 1994). The alerting network is responsible for maintaining response readiness and is commonly divided into two modes; phasic alertness refers to a transient increase in arousal due to the presence of an alerting signal, while tonic alertness represents an intrinsic level of arousal sustained over longer periods (Aston-Jones and Cohen, 2005; Sturm et al., 1999). While an increase in phasic alertness is typically associated with an increase in visual processing speed (Petersen et al., 2017) and a decrease in response times (Hackley, 2009; Posner et al., 1973), it is also associated with increased distractor interference (Tromp et al., 2025).

In the arrow flanker task, participants must respond to the direction (left or right) of a central target arrow flanked by arrows that are pointing in the same (congruent) or opposite (incongruent) direction. Responses are typically faster and more accurate on congruent compared to incongruent trials, a finding referred to as the congruency effect (Eriksen and Eriksen, 1974). The congruency effect indexes distractor interference, with larger effects reflecting reduced attentional selectivity (Botvinick et al., 2001; Gratton et al., 1992). Congruency effects are most often observed in response times, but speed emphasis can shift its expression toward error rates; under speed pressure, participants may respond before incorrect activation is fully suppressed, which can cause congruency effects to appear in the accuracy data even when the mean reaction time (RT) effect is weak or absent (Gratton et al., 1988; Mittelstädt et al., 2022). This pattern is consistent with dual-route accounts in which a fast, automatic route driven by the full stimulus array competes with a slower, deliberative route specific to the target, such that fast responses are disproportionately error-prone on incongruent trials (Ridderinkhof et al., 2020). As such, both RTs and accuracy can offer complementary insight into distractor interference. Surprisingly, given that phasic alertness typically leads to improved cognitive performance, in the Flanker task the congruency effect is larger on trials preceded by an alerting cue compared to trials that are not (Callejas et al., 2005; Schneider, 2019b). This is surprising as one might expect an alert observer to be better, not worse, at filtering irrelevant distractors (Callejas et al., 2005; Weinbach and Henik, 2011).

Here we focus on two accounts that have been proposed to explain this alerting-congruency interaction (others are discussed in the General Discussion). First, alerting cues may serve to disrupt cognitive control (Callejas et al., 2004; Callejas et al., 2005; Tromp et al., 2025), by reducing the ability to filter distractors. Second, alerting may enhance the speed of early stimulus processing and automatic stimulus–response (S-R) mappings (Fischer et al., 2010, 2012), which is supported by ERP data showing that alerting affects S-R translations and strengthens automatic activation of the incorrect response on incongruent trials (Asanowicz et al., 2019; Kottik et al., 2025). In the current study, we investigate whether the alerting-congruency interaction is influenced by characteristics of the preceding trial in order to disentangle these two accounts. If the alterting congruency interaction is driven by variations in cognitive control, this effect may depend on previous trial congruency. In contrast, if the alterting-congruency interaction is driven by variations in automatic response activation, this effect may depend on response repetition.

Congruency sequence effects (CSE) have been reported in the Flanker task where the size of the congruency effect is reduced following incongruent trials relative to congruent trials (Botvinick et al., 2001, 2004; Gratton et al., 1992). This reduction could partly reflect proactive control caused by forward-acting anticipatory upregulation of goal-relevant information when conflict is high, or alternatively transient, retroactive control where adjustments are made following conflict (Braver, 2012). In either situation, control should be high following incongruent trials. However, an entirely different possibility is that the CSE is caused by stimulus–response priming or feature integration (Egner, 2007; Hommel et al., 2004; Mayr et al., 2003; Nieuwenhuis et al., 2006). According to these accounts the CSE does not reflect adjustments in control but instead is driven by an episodic memory process that unfold on a trial-to-trial basis (Mayr et al., 2003; Egner, 2007). When features fully repeat from one trial to the next, RTs are fast and accurate. However, when there is partial repetition, as will happen when the response and the target repeat but the distractor mismatches, RTs and errors will increase because the previous event file must be updated (Hommel et al., 2004). When the response switches, the retrieved event file contains the wrong response, creating conflict that undermines the relevance of matching, so symmetrical patterns across repeat and switch trials are not expected. Mayr et al. (2003) note that the arrow flanker task yields eight possible trial sequences (see Table 1) and stimulus–response priming can account for the CSE on trials with a repeated response. When the response repeats, exact repetitions (CC, II) are especially fast, whereas partial repetitions (CI, IC) are slowed. This pattern artificially enlarges the congruency effect following congruent trials and reduces it following incongruent trials. We note that Mayr et al. (2003) and Nieuwenhuis et al. (2006) describe this effect primarily in terms of stimulus–response priming, where priming effects should be greater on repeat relative to switch trials, whereas Hommel et al. (2004) and Egner (2007) frame this in terms of feature integration. In the present study, we take the approach of Mayr et al. (2003) and Nieuwenhuis et al. (2006) and focus on stimulus–response priming, as response repetition (repeat vs. switch) is included as an independent variable, but we note that this could also be conceptualized as feature integration.

**Table 1:** Trial to trial transitions in the arrow flanker task.

Trial N-1	Trial N	Condition	Response overlap
>>>	>>>	CC	Repeat
>>>	<><	CI	Repeat
<><	>>>	IC	Repeat
<><	<><	II	Repeat
>>>	<<<	CC	Switch
>>>	><>	CI	Switch
<><	<<<	IC	Switch
<><	><>	II	Switch

Prior-trial congruency and response repetition provide distinct leverage points for examining the two theoretical accounts. If alerting cues disrupt cognitive control, the alerting-congruency interaction should be larger following incongruent trials than congruent trials, regardless of whether the response repeats or switches. If instead alerting enhances automatic S-R activation, then the interaction should be larger on response-repeat relative to response-switch trials regardless of prior trial congruency.

We note that Soutschek et al. (2013) and Liu et al. (2013) have also examined sequential effects on alerting in conflict tasks, but they did so in ways that do not test whether prior-trial congruency and response repetition moderate the alerting-congruency interaction. Specifically, Soutschek et al. (2013) manipulated nonpredictive accessory stimuli in Simon and Stroop tasks in a blockwise manner, while excluding repetition trials. The blockwise design means that alertness level was held constant across entire blocks rather than varying trial-by-trial, which affects the extent to which trial-by-trial fluctuations in phasic alertness can be examined. Furthermore, by excluding repetition trials, Soutschek et al. (2013) could not assess whether response repetition moderates the relationship between alerting and congruency, which is a central question here. Liu et al. (2013) correlated individual differences in a measure of alerting efficiency to sequential effects in a letter flanker task. While informative about how chronic differences in alertness relate to cognitive control, this approach cannot speak to how a phasic alerting cue on a given trial interacts with congruency or response repetition. In contrast, the present study is the first to test within the same arrow-flanker task whether the alerting-congruency interaction is moderated by previous-trial congruency and response repetition.

If the alerting-congruency interaction depends on the level of control engaged during the flanker task, it may vary with working memory capacity (WMC). WMC reflects a person’s ability to maintain task goals and control attention in situations where distraction is present, with some individuals exerting large amounts of cognitive control and others responding in a more reflexive manner (Engle and Kane, 2004). If alerting signals disrupt control then the alerting-congruency interaction might be stronger for individuals who have relatively greater levels of control. One way to assess this is with an automated operation span task (AOSPAN) (Unsworth et al., 2005). Redick and Engle (2006) used this task to examine the extent to which individuals with relatively high or low WMC differ in their performance on the Attention Network Test (ANT; Fan et al., 2002). They found that WMC affected the amount of interference in the control network but did not impact the other attention networks. The current study seeks to determine if AOSPAN is positively correlated with the alerting-congruency interaction, where higher span individuals are more strongly affected by alerting cues. We note however that Patterson and Kahan (2022) examined whether a verbal working memory load affects the alerting-congruency interaction, and results show that a memory load of this sort slows RTs but does not moderate the interaction. However, working memory may involve multiple components (Baddeley, 1986, 2007) and a verbal memory load may occupy resources in one component (e.g., the phonological loop) rather than impacting cognitive control. As such, it remains possible that a complex span task that assess the ability to maintain focus in the presence of distraction may correlate with the magnitude of the alerting-congruency interaction.

The current experiment tests whether preceding trial congruency or response repetition moderate the alerting-congruency interaction. If phasic arousal induced by an alerting cue disrupts control, then the alerting-congruency interaction should be larger when control is the highest (following incongruent rather than congruent trials, regardless of response repetition). In addition, the size of this interaction should be positively correlated with AOSPAN scores. If instead phasic arousal speeds early stimulus processing and automatic S-R mappings, the alerting-congruency interaction should be larger on response-repeat than response-switch trials, and should not correlate with AOSPAN scores. Of course, it is also possible that the alerting-congruency interaction will be moderated by both, or neither, type of preceding trial.

## Methods

### Participants

We used MorePower 6.0.4 (Campbell and Thompson, 2012) to estimate our required sample size. Because our key question concerned whether the alerting-congruency interaction was moderated by the preceding trial type (i.e., a three-way interaction) and whether the alerting-congruency interaction was correlated with AOSPAN (i.e., a Pearson Correlation), power analyses indicated that detecting a medium-to-large three-way interaction ( $\eta_{p}^{2}$ = 0.06–0.11) or correlation (*r* = 0.30–0.50) with 0.95 power required approximately 120–206 and 44–136 participants, respectively. To ensure adequate power, we recruited 181 individuals through CloudResearch’s Connect platform, each of whom was paid $8 to complete the study online. Participants whose accuracy scores were two or more standard deviations below the mean were excluded, resulting in the removal of 16 of the 181 participants. In addition, one participant had extremely long reactions times (over 8 SD from the mean) and this individual was also dropped. Among the remaining 164 participants, 72 identified as women, 91 as men, and 1 did not report their gender. One woman indicated that she identifies as transgender. The average age of the sample was 40 years, with ages ranging from 19 to 78.^1^

### Procedure

The study began in Qualtrics with an online informed consent. Qualtrics then randomly determined the order in which participants completed the two tasks (arrow flanker or AOSPAN), redirecting them to each in turn. Each task opened in a new window, where participants were asked to paste their unique subject number. For both tasks, participants watched a video describing the procedure and they could not begin until the video finished. The videos were followed by duplicate on-screen written instructions. The arrow-flanker task was programmed in OpenSesame (Mathôt et al., 2012) and was generated for web-based testing using PsychoPy in the back-end layer (Peirce, 2007); this was hosted on a JATOS server (Lange et al., 2015). AOSPAN was programmed in JavaScript by Luthra and Todd (2019). The experiment lasted approximately 35–40 min.

#### Arrow-flanker task

**Figure 1:**
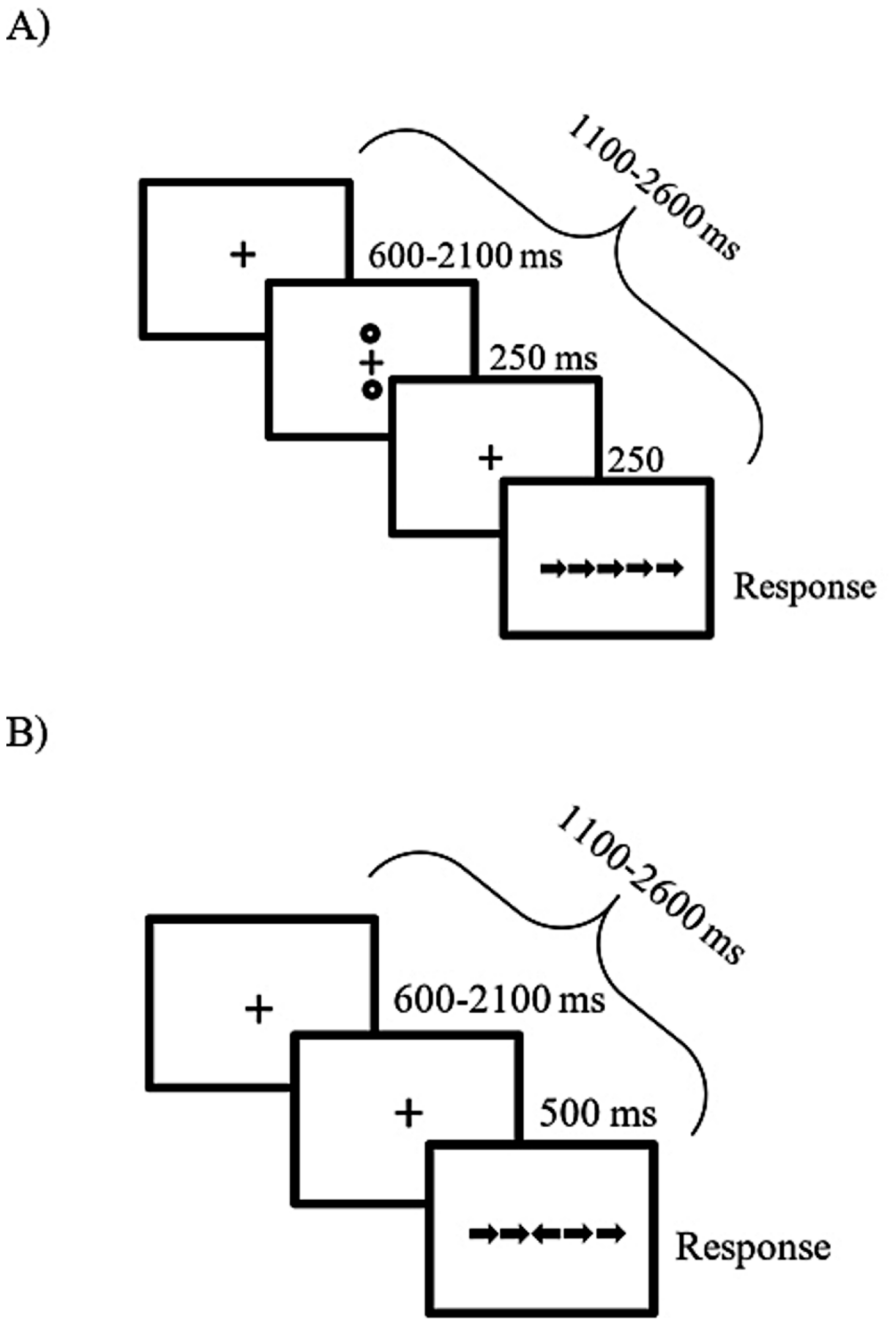
Timing of events in the arrow-flanker task in the alerting **(A)** and no alerting **(B)** conditions. **(A)** Shows a congruent trial and **(B)** shows an incongruent trial.

On each trial in the arrow-flanker task, a centrally presented fixation (+) appeared in 18-point Arial Black font. This fixation display remained on the screen for a randomly determined inter-trial interval (ITI) ranging from 1,100 to 2,600 ms in 100-ms increments. This was followed by the appearance of the target-and-distractor display. We kept a variable ITI to mirror other studies that have examined the alerting-congruency interaction (e.g., Schneider, 2018). The target-and-distractor display consisted of five arrows: four flanking distractor arrows surrounding a central target arrow. The flanking arrows all pointed in either the same (congruent) or opposite (incongruent) direction as the target, and participants were instructed to respond to the direction of the target by pressing the Q and P keys for left and right, respectively. The direction of both the target and distractor arrows was randomly determined on a trial-by-trial basis. On half of the trials, an alerting cue appeared 500 ms before the onset of the target-and-distractor display (Figure 1A), and on the other half, no alerting cue was shown (Figure 1B).^2^ The alerting cue consisted of two circles (16 pixels in diameter) positioned above and below the fixation (64 pixels in each direction), and provided no spatial information about the target. Each of the arrows measured 50 pixels in length and the arrow head measured 30 pixels across. Example sequences are shown in Figure 1.

Participants completed 8 practice trials followed by 10 blocks of 32 experimental trials (i.e., 320 total trials). Because all manipulated factors varied in a fully randomized manner, the exact number of trials of each type could vary slightly across participants. As such, the proportion of congruent and incongruent trials could vary slightly from 50%.^3^ Participants were given RT and accuracy feedback after practice, but none during experimental trials. Between each block, participants could take self-paced breaks.

#### AOSPAN task

The AOSPAN task was modeled after Unsworth et al. (2005) and was programmed in JavaScript by Luthra and Todd (2019).^4^ On each trial, letters (from the set: F, H, J, K, L, N, P, Q, R, S, T, V) were presented centrally for 1,000 ms each. Each letter was followed by a simple math problem (e.g., 9 + 3 = 12), which remained on the screen until participants verified its accuracy (true or false) or until 6 s elapsed. Each trial contained 4–7 letters, with three displays per set size (12 total), presented in random order. After each set, participants saw 12 letters arranged in a 4 × 3 grid. Participants used the mouse to select the letters in presentation order and the selected letters appeared at the top of the screen. A button at the bottom of the screen allowed them to correct errors, and another button allowed them to submit their final response. The AOPSAN task started with 3 sets of practice letters that were not interspersed with math problems, followed by 5 sets of math problems that were not interspersed with letters. Following practice trials, the 12 critical trials were presented.

## Results

Accuracy rates and RTs were recorded in the 16 conditions created from the factorial crossing of response repetition (repeat vs. switch), previous trial congruency (congruent vs. incongruent), alerting condition (cued vs. not cued), and current trial congruency (congruent vs. incongruent). When calculating average RTs, only trials that were responded to correctly were included. To lessen the impact of unusually long outliers while keeping RTs on the same millisecond scale, we analyzed geometric means. For each of the 16 conditions, we took the log of each correct-trial RT, averaged those log values, and then converted the result back to milliseconds by taking the antilog (see Kahan and Zhang, 2019; Patterson and Kahan, 2022).^5^

**Figure 2:**
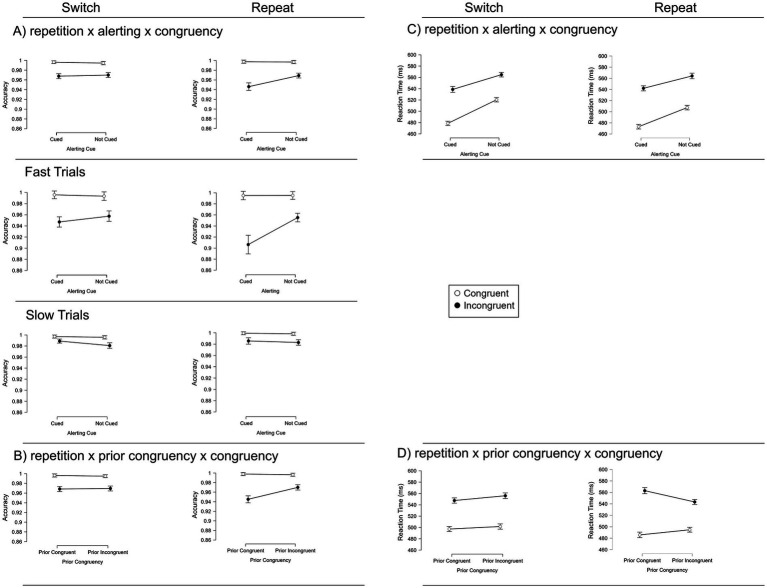
Accuracy **(A,B)** and reaction times **(C,D)** in the arrow flanker task. **(A,C)** Depict the repetition × alerting × congruency interaction; **(B,D)** depict the repetition × prior congruency × congruency interaction. The left side of each panel shows switch trials; the right side of each panel shows repeat trials. Error bars reflect 95% CIs.

Analyses were conducted in JASP (JASP Team, 2025). Accuracy and RTs were analyzed using a 2 (response repetition: repeat vs. switch) × 2 (previous trial congruency: congruent vs. incongruent) × 2 (alerting: cued vs. not cued) × 2 (current trial congruency: congruent vs. incongruent) repeated-measures ANOVA. Full ANOVA summaries are reported in Table 2. Here we focus on the critical three-way interactions of interest along with follow-up analyses. For the accuracy data there was a response repetition × alerting × congruency interaction (*p* < 0.001; Figure 2A) but no four-way interaction with previous trial congruency (*p* = 0.357). Given that previous trial congruency did not moderate the 3-way interaction, data were collapsed across this factor to test whether the alerting × congruency interaction differs as a function of response repetition. For response-switch trials, only the main effect of congruency was significant, *F*(1, 163) = 55.98, *p* < 0.001, $\eta_{p}^{2}$ = 0.26; the alerting × congruency interaction was not, *F*(1, 163) = 0.95, *p* = 0.332, $\eta_{p}^{2}$ = 0.01 (Figure 2A left). To quantify evidence for the null hypothesis, a Bayesian 2×2 ANOVA was conducted on response-switch trials. The model receiving the strongest support included a main effect of congruency (BF₁₀ = 1.73 × 10^9^). A competing model that included the alerting × congruency interaction was less supported (BF₁₀ = 3.29 × 10^7^). Direct comparison of these Bayes factors (Wagenmakers et al., 2018) indicates that the data are 52.58 times more likely under the model without the alerting × congruency interaction. However, when the response on the preceding trial repeated, the alerting × congruency interaction was significant, *F*(1, 163) = 29.07, *p* < 0.001, $\eta_{p}^{2}$ = 0.15 (Figure 2A right). A direct comparison of repeat and switch trials in the cued-incongruent condition showed lower accuracy on repeat trials, *t*(163) = 5.19, *p* < 0.001, as expected if alerting cues increase incorrect response activation on incongruent trials. To further test the possibility that alerting cues and response repetition combine to enhance early motor activation, which increases the likelihood of making fast but premature responses, we conducted an analysis of fast vs. slow responses. To do this we used a median spilt of each person’s RT in each condition. These data were analyzed in a speed × response repetition × alerting × congruency ANOVA. The four-way interaction was significant, *F*(1, 163) = 9.21, *p* = 0.003, $\eta_{p}^{2}$ = 0.05, such that the three-way interaction on fast trials was significant *F*(1, 163) = 16.47, *p* < 0.001, $\eta_{p}^{2}$ = 0.09 (see Figure 2A-fast), but the three-way interaction on slow trials was not, *F*(1, 163) = 0.92, *p* = 0.338, $\eta_{p}^{2}$ = 0.01 (see Figure 2A-slow).^6^

**Table 2:** Summary table for the 2 × 2 × 2 × 2 repeated measures ANOVA on the accuracy and RT data.

ANOVA	Source of variation	SS	df	MS	F	*p*	$\eta_{p}^{2}$	Figure 2 panel
Acc	Rep	0.015	1	0.015	8.714	0.004	0.051	
	Residuals	0.288	163	0.002				
	Prior Cong	0.022	1	0.022	19.118	< 0.001	0.105	
	Residuals	0.183	163	0.001				
	Cong	0.719	1	0.719	75.787	< 0.001	0.317	
	Residuals	1.547	163	0.009				
	Alerting	0.02	1	0.02	14.615	< 0.001	0.082	
	Residuals	0.228	163	0.001				
	Rep * Prior Cong	0.023	1	0.023	25.467	< 0.001	0.135	
	Residuals	0.15	163	9.194 × 10^−4^				
	Rep * Cong	0.028	1	0.028	22.076	< 0.001	0.119	
	Residuals	0.204	163	0.001				
	Prior Cong * Cong	0.034	1	0.034	32.075	< 0.001	0.164	
	Residuals	0.175	163	0.001				
	Rep * Alerting	0.019	1	0.019	19.12	< 0.001	0.105	
	Residuals	0.162	163	9.933 × 10^−4^				
	Prior Cong * Alerting	3.279 × 10^−6^	1	3.279 × 10^−6^	0.003	0.956	1.894 × 10^−5^	
	Residuals	0.173	163	0.001				
	Cong * Alerting	0.031	1	0.031	20.769	< 0.001	0.113	
	Residuals	0.246	163	0.002				
	Rep * Prior Cong * Cong	**0.023**	**1**	**0.023**	**20.844**	**< 0.001**	**0.113**	**B**
	Residuals	0.182	163	0.001				
	Rep * Prior Cong * Alerting	0.002	1	0.002	2.032	0.156	0.012	
	Residuals	0.175	163	0.001				
	Rep * Cong * Alerting	**0.015**	**1**	**0.015**	**13.935**	**< 0.001**	**0.079**	**A**
	Residuals	0.179	163	0.001				
	Prior Cong * Cong * Alerting	5.338 × 10^−4^	1	5.338 × 10^−4^	0.476	0.491	0.003	
	Residuals	0.183	163	0.001				
	Rep * Prior Cong * Cong * Alerting	6.652 × 10^−4^	1	6.652 × 10^−4^	0.854	0.357	0.005	
	Residuals	0.127	163	7.790 × 10^−4^				
RTs	Rep	9997.16	1	9997.16	2.61	0.108	0.016	
	Residuals	624288.69	163	3829.99				
	Prior Cong	184.95	1	184.95	0.289	0.591	0.002	
	Residuals	104236.22	163	639.49				
	Cong	2.176 × 10^+6^	1	2.176 × 10^+6^	627.746	< 0.001	0.794	
	Residuals	565025.99	163	3466.42				
	Alerting	635016.98	1	635016.98	296.878	< 0.001	0.646	
	Residuals	348653.99	163	2138.98				
	Rep * Prior Cong	22846.95	1	22846.95	35.636	< 0.001	0.179	
	Residuals	104501.34	163	641.11				
	Rep * Cong	18245.56	1	18245.56	18.066	< 0.001	0.1	
	Residuals	164622.87	163	1009.96				
	Prior Cong * Cong	24913.42	1	24913.42	24.707	< 0.001	0.132	
	Residuals	164359.68	163	1008.34				
	Rep * Alerting	5358.44	1	5358.44	4.654	0.032	0.028	
	Residuals	187691.82	163	1151.48				
	Prior Cong * Alerting	16.5	1	16.5	0.016	0.899	9.978 × 10^−5^	
	Residuals	165340.84	163	1014.36				
	Cong * Alerting	31635.44	1	31635.44	32.54	< 0.001	0.166	
	Residuals	158,470	163	972.21				
	Rep * Prior Cong * Cong	**43587.03**	**1**	**43587.03**	**35.119**	**< 0.001**	**0.177**	**D**
	Residuals	202301.59	163	1241.11				
	Rep * Prior Cong * Alerting	138.82	1	138.82	0.09	0.765	5.495 × 10^−4^	
	Residuals	252481.05	163	1548.96				
	Rep * Cong * Alerting	**520.72**	**1**	**520.72**	**0.497**	**0.482**	**0.003**	**C**
	Residuals	170893.77	163	1048.43				
	Prior Cong * Cong * Alerting	3708.37	1	3708.37	2.714	0.101	0.016	
	Residuals	222714.49	163	1366.35				
	Rep * Prior Cong * Cong * Alerting	191.48	1	191.48	0.282	0.596	0.002	
	Residuals	110527.21	163	678.08				

Type III Sum of Squares; Acc, accuracy; RT, Reaction Time; Rep, Response Repetition; Prior Cong, Previous Trial Congruency; Cong, Current Trial Congruency; Alerting, Alerting Condition. Critical effects appear in bold and the Figure 2 panel letter is shown.

The data also show that the alerting × congruency interaction was not moderated by previous trial congruency (*p* = 0.491) and as such there is no evidence that the alerting × congruency interaction is affected by cognitive control. Instead, we find a response repetition × previous trial congruency × congruency interaction (*p* < 0.001; Figure 2B). This parallels findings by Mayr et al. (2003) and Nieuwenhuis et al. (2006) who report that stimulus–response priming plays a critical role in the arrow-flanker task. When data from switch and repeat trials are analyzed separately, the previous trial congruency × congruency interaction is significant for repeat trials, *F*(1, 163) = 43.78, *p* < 0.001, $\eta_{p}^{2}$ = 0.21 (Figure 2B right), but not switch trials, *F*(1, 163) = 1.29, *p* = 0.258, $\eta_{p}^{2}$ = 0.01 (Figure 2B left).

For the RT data the response repetition × alerting × congruency interaction was not significant (*p* = 0.482; Figure 2C)^7^ nor was the previous trial congruency × alerting × congruency interaction (*p* = 0.101). As was the case with accuracy rates, the response repetition × previous trial congruency × congruency interaction was significant (*p* < 0.001; Figure 2D). To better understand this, when the data from switch and repeat trials are analyzed separately, the previous trial congruency × congruency interaction is significant for repeat trials, *F*(1, 163) = 47.33, *p* < 0.001, $\eta_{p}^{2}$ = 0.23 (Figure 2D right), but not switch trials, *F*(1, 163) = 1.50, *p* = 0.222, $\eta_{p}^{2}$ = 0.01 (Figure 2D left). As such the RT data provide no evidence of increased control following incongruent trials, since this type of adjustment should have appeared on both switch and repeat trials.

For the AOSPAN task, we calculated each participant’s total number of letters recalled in the correct position, which could range from 0 to 66. We also computed each participant’s accuracy in verifying the math problems presented during the task. AOSPAN scores (M = 51.5, range = 2–66) were then correlated with math-accuracy rates (M = 0.97), as well as with the magnitude of the alerting-congruency interaction. As expected, AOSPAN scores were positively correlated with math performance, *r*(162) = 0.33, *p* < 0.001, indicating that participants who engaged more fully with the AOSPAN task performed better in both the memory and math portions of the task. AOSPAN scores were not correlated with the magnitude of the alerting × congruency interaction for either accuracy, *r*(163) = 0.06, *p* = 0.442, or response time, *r*(163) = −0.04, *p* = 0.581. Bayesian analyses further support the absence of an association as the data were more likely under the null hypothesis (BF₀₁ = 7.63 for accuracy; BF₀₁ = 8.80 for RT) than under the alternative.

## Discussion

The data are more broadly consistent with the view that alerting enhances S-R activation than disrupting cognitive control. First, if alerting and response repetition have a synergistic effect to enhance early motor activation, then this might increase premature commitment to incorrect responses on incongruent trials. Consistent with this, the alerting × congruency interaction in accuracy was moderated by response repetition. However, the RT analysis did not show this moderating effect, so while repetition may enhance the interaction, it is not required. Despite this, we believe the differing patterns are interpretable if speed shifts the expression of conflict from RTs to early error dynamics (Mittelstädt et al., 2022; Wylie et al., 2009; van der Lubbe et al., 2025), which may occur if incongruent flankers produce rapid incorrect response activation as reported elsewhere (Gratton et al., 1988; Stins et al., 2007). This is also consistent with the observation that the alerting × congruency × repetition interaction was strongest for fast responses. Additionally, the alerting × congruency interaction did not depend on previous trial congruency for either measure. Together, we believe that the moderating effect of response-repetition on fast trials in the accuracy data, the absence of modulation by previous-trial congruency in either measure, and the Bayesian evidence of no association with AOSPAN, challenge accounts that phasic arousal inhibits cognitive control (Callejas et al., 2004, 2005), and are more consistent with the view that phasic arousal enhances stimulus–response activation (Asanowicz et al., 2019; Fischer et al., 2010, 2012).

Critically, if phasic alertness disrupted cognitive control, then the alerting-congruency interaction should have been amplified following incongruent trials, where control is presumed to be high. This pattern was not observed. Moreover, individual differences in working memory capacity, as measured by AOSPAN, were unrelated to the size of the interaction. If alerting cues impaired cognitive control, one might expect larger disruptions among individuals who typically exert greater control. The absence of such a relationship further favors an account centered on enhanced automatic response activation rather than impairments in top-down control. Future studies examining the alerting-congruency interaction should therefore include response repetition as a factor, as failing to do so may obscure theoretically informative patterns.

That said, there was no compelling evidence that cognitive control varied as a function of previous trial congruency in this experiment, as the congruency sequence effect was restricted to response-repetition trials. This pattern is consistent with a stimulus–response priming account. As such, it remains possible that in a paradigm where cognitive control fluctuates, variations in control could modulate the alerting-congruency interaction. Thus, the current findings should not be taken as evidence that phasic arousal can never disrupt control, only that this was not supported under the present conditions.

We note that we did not observe a four-way interaction among alerting, congruency, response repetition, and prior-trial congruency in the accuracy or RT data. If alerting cues (i.e., phasic arousal) enhanced S-R binding, one might predict a larger repetition × prior congruency × congruency interaction, which has been attributed to episodic binding (Mayr et al., 2003; Nieuwenhuis et al., 2006), to be greater on cued relative to un-cued trials (i.e., a four-way interaction). However, it is entirely plausible that phasic arousal does not affect S-R binding. Instead, we believe the data indicate that phasic arousal strengthens response-code activation, and speculate that response repetition has two separate effects. First, repetition might enhance episodic retrieval, which results in the repetition × prior congruency × congruency interaction (Mayr et al., 2003; Nieuwenhuis et al., 2006). Second, repetition may increase reliance on motor codes at a fast-acting pre-decisional stage (c.f., Kunde et al., 2003; Schmidt et al., 2011). If these processes were to co-occur and operate on distinct representational systems (one motoric and the other involving episodic retrieval) then a four-way interaction would not be expected. This speculative account awaits further testing.

The proposal that phasic arousal boosts automatic response activation is further supported by findings that this interaction emerges primarily when stimuli have well-established directional associations. The effect is reliably observed with arrows (Schneider, 2018a, 2018b), Simon tasks (Böckler et al., 2011) spatial Stroop tasks (Kahan and Smith, 2024), and numerical magnitude judgments that map onto space (the SNARC effect; Kahan and Zhang, 2019). By contrast, it is often absent in tasks whose stimuli lack such associations, like the Stroop task (Schneider, 2019b). This pattern further supports the claim that phasic arousal boosts the activation of well-established response codes.^8^

Other theoretical perspectives have proposed that alerting widens the spatial focus of attention (Weinbach and Henik, 2012) or biases processing toward perceptually salient features (Weinbach and Henik, 2014). While these mechanisms may operate in some contexts, they do not readily account for why the alerting-congruency interaction that was observed here depended on response repetition. A generalized broadening of attention or salience bias should have influenced switch trials as well as repeat trials. Similarly, the early onset hypothesis (Nieuwenhuis & de Kleijn, 2013), which proposes that alerting reduces stimulus-encoding to a point when cognitive control is still developing cannot straightforwardly predict why the effect would emerge selectively on response-repetition trials but not on response-switch trials. Our results therefore strengthen the interpretation that phasic arousal amplifies prepotent stimulus–response associations.

Taken together, the present findings elucidate how phasic arousal affects performance in the presence of response conflict. When individuals are given an alerting cue, the perceptual and response systems become rapidly engaged. This readiness appears to accelerate the activation of stimulus–response mappings, increasing the likelihood that an automatically primed response will dominate behavior. As such, the phasic arousal that is caused by an alerting cue comes at the potential cost of strengthening automatically activated response pathways, thereby impairing performance when distracting stimulus–response associations conflict with task goals.

## Data Availability

The datasets presented in this study can be found in online repositories. The names of the repository/repositories and accession number(s) can be found at: https://osf.io/xefc3/overview?view_only=fead7c5780a84567912a3f0eb4167be0.

## Footnotes

1 Data from all 181 participants are available on the OpenScience framework. See: https://osf.io/xefc3/overview?view_only=fead7c5780a84567912a3f0eb4167be0

2 The 500-ms cue–target interval matches prior studies (e.g., Kahan and Zhang, 2019), though the alerting effects observed here may reflect either exogenous or endogenous attentional shifts (Lawrence and Klein, 2013).

3 By manipulating trial type in a fully randomized manner expectations regarding the upcoming stimulus could not be formed in advance. The number of trials in each of the 16 conditions is posted on the OpenScience framework and this did not vary greatly across participants with a mean of 20 and a standard deviation of 4.

4 The AOSPAN task can be downloaded from: https://github.com/mahiluthra/working_memory_tests

5 Because some readers may be less familiar with geometric means, which have the advantage of retaining all RTs rather than arbitrarily excluding some values, we also conducted a trimming analysis, removing RTs more than 3 SDs from each participant’s mean in each condition. This analysis produced the identical pattern such that all significant effects remained significant, and no new effects emerged.

6 We note that the three-way interaction in Figure 2A occurs near the top of the scale, raising the possibility that a ceiling effect might explain the pattern. We do not believe it does. If we focus on incongruent trials alone, which are demonstrably below ceiling, accuracy on repeat-not-cued-incongruent trials is significantly worse than repeat-not-cued-congruent trials, *t*(162) = 7.63, *p* < 0.001. Restricting the analysis in Figure 2A to incongruent trials (solid data points) in a 2 (alerting) × 2 (repetition) ANOVA still yields a significant interaction, *F*(1, 163) = 19.67, *p* < 0.001. Specifically, switch and repeat trials do not differ when not cued, *t*(163) = 0.07, *p* = 0.946, but do differ significantly when cued, *t*(163) = 5.19, *p* < 0.001. This is precisely as predicted if alerting cues have a synergistic effect with response repetition to increase incorrect response activation on incongruent trials. Furthermore, because the predicted pattern is even stronger on fast trials, where performance is lowest, a ceiling explanation becomes even less tenable.

7 We note that incongruent trials with a repeated response and alerting cue were responded to more rapidly than those with no cue, *t*(163) = 7.31, *p* < 0.001, yet accuracy was lowest under these same conditions (see Figure 2A). This pattern is consistent with dual-process models in which a highly active, premature, incorrect response is acted upon before slower control processes can suppress it (Ridderinkhof et al., 2020).

8 Although the alerting-congruency interaction is often absent in the Stroop task, our lab has evidence that this interaction can emerge under conditions that speed the distractor’s response activation (Golden et al., 2025). In particular, when the distractor is given a head start we find that phasic arousal will boost the Stroop effect, which is consistent with the results of the current study where alerting effects are most evident when response activation is primed (e.g., on response-repetition trials).
